# Assessment of ethnobotanical uses, household, and regional genetic diversity of aroid species grown in northeastern India

**DOI:** 10.3389/fnut.2023.1065745

**Published:** 2023-03-31

**Authors:** Veerendra Kumar Verma, Amit Kumar, Heiplanmi Rymbai, Hammylliende Talang, Priyajit Chaudhuri, Mayanglambam Bilashini Devi, Nongmaithem Uttam Singh, Samrendra Hazarika, Vinay Kumar Mishra

**Affiliations:** ICAR Research Complex for NEH Region, Umiam, Meghalaya, India

**Keywords:** aroid, taro, genetic diversity, starch, oxalic acid, molecular marker

## Abstract

Aroids are an important group of indigenous tuber crops, grown widely for their leaves, petioles, stolons, corms, and cormels. A total of 53 genotypes were evaluated for their genetic diversity in northeastern region of India. At household level, a total of 16 landraces of Aroids were recorded having different ethnobotanical uses. Based on the population study under *Jhum*/Shifting farming, landrace Rengama was dominant in area with 47% of the total population followed by Tamachongkham and Tasakrek. However, Pugarkusu and Chigi occupied 33.0 and 24.0% of the population, respectively under backyard farming, and were considered as major landraces. Tamachongkham, high in acridity and total oxalate content (0.82%), was used for cooking with meat, while Tasakrek was used as a baby food due to high total sugar (>3.0%), low in acridity, and total oxalate content (<0.12%). The Simpson’s diversity index of the backyards was higher (0.80) as compared to *Jhum* field (0.63). The genotypes showed wider variability in growth and yield attributes like; plant height (89.4–206.1 cm), number of side shoots (1.84–5.92), corm weight (38.0–683.3 g), cormel weight (14.0–348.3 g), yield (0.24–1.83 kg plant^−1^). Similarly, wide variations were also observed for quality traits like total sugar (1.93–4.94%); starch (15.32–32.49%), total oxalate (0.10–0.82%), and dry matter (16.75–27.08%) content. Except for total oxalate, all the growth and yield attributes have shown high heritability and moderate to high genetic advance. Molecular analysis (33 polymorphic SSR markers) detected a total of 136 alleles, ranged 3 to 8 alleles per marker. The observed heterozygosity (0.24) was less than expected heterozygosity (0.69). The group-wise maximum genetic divergence was observed between *Colocasia fallax* (cv. Chigi) to *C. esculenta* var. *aquatilis* (cv. Tharsing); *C. fallax* (*cv*. Chigi) to *C. gigantea* (*cv*. Ganima) and *C. gigantea* (*cv*. Ganima) to *Xanthosoma* spp., while it was least between eddo and dasheen. The findings indicated, a wider diversity and distinct ethnobotanical uses of Aroid landraces at the house hold levels, which should be conserved and popularized to ensure nutritional security.

## Introduction

Northeastern states of India are one of the important hotspots of the world’s biodiversity. Due to humid subtropical climate, there is a wide range of cultivated and wild plant species available in the region. The majority of the Aroid species are native to the region, and belong to Araceae family; among them *Colocasia esculenta* var. *antiquorum* (Arvi/Eddoe), *Colocasia esculenta* var. *esculenta* (Bunda/Dasheen), *Colocasia esculenta* var. *aquatilis* (edible stolon), *Colocasia fallax* (edible leaves and petioles), *Xanthosoma sagittifolium* (Cocoyam/Tannia for edible petioles), and wild Giant taro (*Alocasia* spp.) are basically used by the farmers for consumption. Taro is considered as one of the world’s oldest food crops, dating back over 9,000 years and was domesticated first in South-East Asia and spread throughout the globe (1). Due to wider adaptability of this crop to diverse climatic conditions, in the present scenario, it is being cultivated worldwide ranging from equatorial tropical to southern and northern temperate zones and rank 5th most consumed root crops in the world (2, 3). Aroids have immense potential to play an important role in ensuring nutritional security, as they are rich sources of carbohydrates, proteins, minerals and vitamins. The tubers are rich in starch (21.2%), protein (3.2%), and minerals like calcium (31.0 mg), magnesium (106 mg), and potassium (356 mg) content. Besides, leaves are also rich in protein (4.98 g), and minerals like calcium (107 mg), potassium (648 mg), phosphorous (60 mg), and vitamins like carotene (2,895 ug), folate (126 ug), and vitamin K (108.6 μg) in 100 g FW (4) and could be utilized to ensure the availability of all the nutrients in a balanced manner. It is also one of the finest sources of dietary fiber (4.1%). Proper cooking, removes the acridity causing factor, calcium oxalate. There are different aroid landraces grown in the region for specific plant parts and uses, like tubers for breakfast, baby food, curry preparation and pickles; leaf and petiole for cooking, pig feed, and also as an ornamental foliage.

Aroids have ethnopharmacological importance, as cooked vegetable contains mucilage and is an effective nervine tonic (5). Leaf juice is a stimulant, expectorant, astringent, appetizer, and otalgia. The juice expressed from the leaf stalks with salt is used as an absorbent in cases of inflamed glands and buboes. Decoction of the peel is used as a folk medicine to cure diarrhea. The juice of the corm is used in cases of alopecia. Internally, it acts as a laxative, demulcent, anodyne, galactagogue and is used in cases of piles and congestion of the portal system, as well as an antidote to the stings of wasps and other insects (6). The crops are also possessing several pharmacological properties like, hypoglycemic, hypolipidemic, anti-inflammatory, antifungal, and anticancerous properties (7–12).

The crop is cultivated widely under different ecology by the tribes of the region. In Meghalaya, over 82.5% of the total cropped area is covered by marginal and small farmers, with <2.0 ha land holding. Over 80% of the population is dependent on agriculture for their livelihood and nutritional security (Government of Meghalaya, India). As, rice is the staple food of the population, farmers have to explore other options in order to increase the nutritional quality of their food and thus, aroids, being a native crop of this region, can play an important role in augmenting nutritional quality. Among the tuber crops, aroid species are grown widely and diverse genetic resources are being cultivated under *Jhum,* as well as a backyard farming system for the year round production and uses*. Jhum* farming is a traditional farming practice followed in entire northeastern hill region of the India except Sikkim. The crops are grown under mixed cropping with chilies, brinjal, okra, ginger, taro, sweet potato, yam (near burnt trees/shrubs), tapioca, cucumber, and rice bean on fences after clearing the forest and burning the dried biomass, especially on the hill slopes. This practice is also known as ‘slash and burn’ or ‘fire-fallow cultivation. In spite of the tremendous potential, the productivity of this crop is meager in the state. It might be due to the rainfed farming especially in the hills, poor quality of planting materials, and poor crop management.

Although, the crop is rich in nutritional value, it is considered as an Orphan crop, and there is no systematic study on the uses and extent of diversity within and between the aroid species in the region. Aroid species are commercially propagated vegetatively using clones (corms, cormels etc.), and most of the species, especially *Colocasia* spp. grown in the region are diploid, with some triploid species (13). Due to poor in flowering and fertility, majority of the varieties developed in the country are the products of clonal selection. Hence for crop improvement through clonal selection with desirable traits (high in yield, starch, dry matter content and low in oxalate), estimation of genetic diversity, and genetic parameters are very important.

There are many approaches for the estimation of genetic diversity like quantitative traits, isozyme and DNA markers; and among them DNA markers have been found most reliable as these markers are reproducible and free from effects of environmental factors. DNA markers has been deployed in many crop species, but in aroid, there are very few reports of utilizing biochemical and different generations of molecular markers, namely, isozyme (14, 15); AFLP (16, 17); RAPD (18, 19); ISSR (20); SSR (21–23) and SNP (24, 25) for assessment of genetic diversity, as well as evaluation of stability (26).

Keeping above in view, the present study was aimed to assess the ethnobotanical uses and genetic diversity of Aroids in the region as well as at household level grown by the tribes, and its potential utilization as an important food crop. This study will be useful in the taxonomic studies, identification of the superior genotypes having unique uses, their characteristics, promotion for commercial production to ensure nutritional and livelihood security, conservation, and utilization in future improvement program.

## Materials and methods

### Survey and collection of germplasm

**Figure 1:**
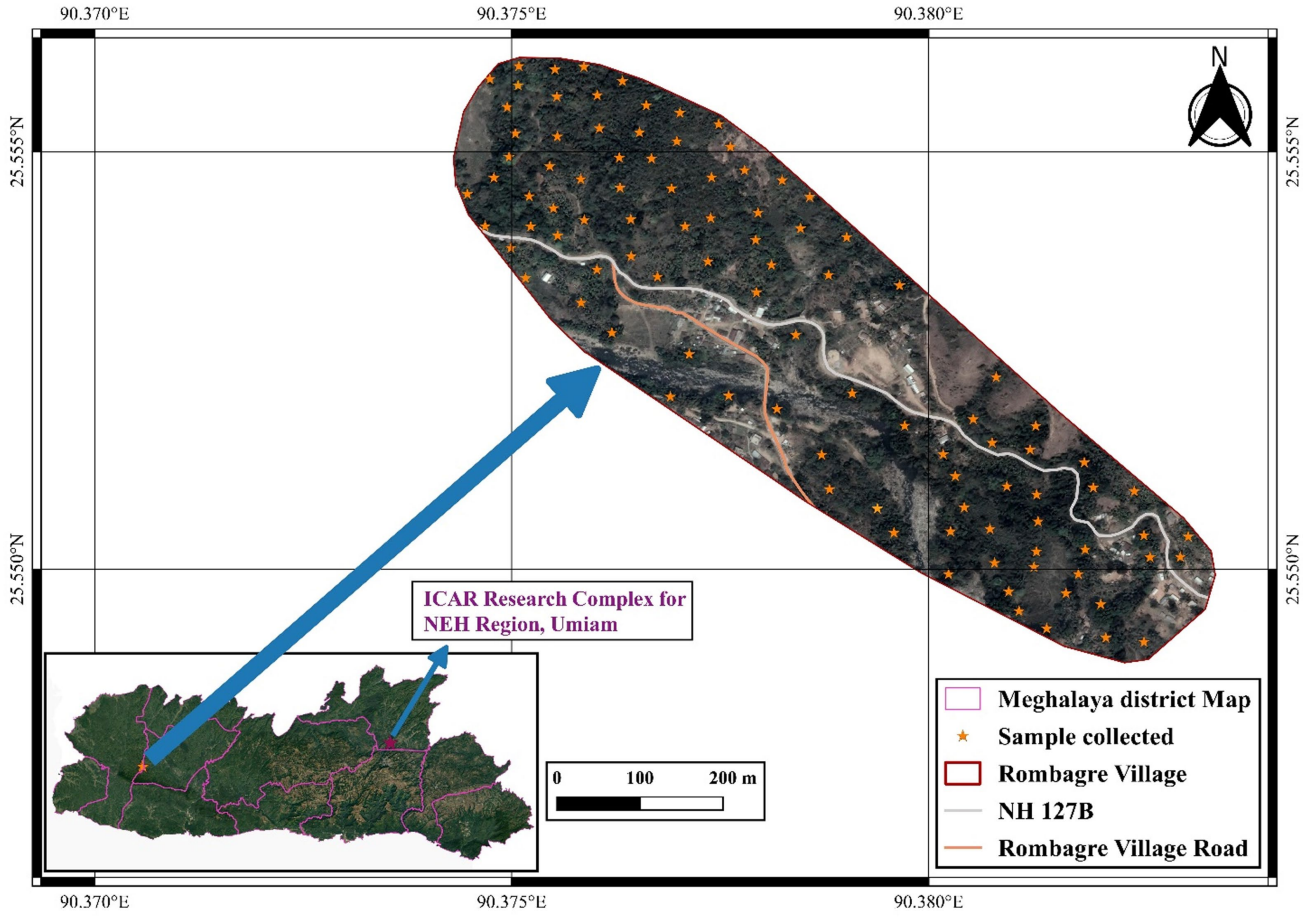
Site of Collection of Aroid species in West Garo Hills of Meghalaya (Altitude: 500–600 m above mean sea level).

To study the diversity of aroids at household level, survey was carried out at the project site, i.e., Rombagre village, West Garo Hills District of Meghalaya, India located under humid subtropics and mid-hill (altitude ranges 500–600 m above mean sea level) conditions (Figure 1). A total of 16 landraces belonging to different species/groups with common variants were observed at each household having distinct ethnobotanical uses (Table 1). The population diversity of the landraces was studied at farmers’ field under *Jhum* as well as backyard of the villagers at household levels covering the entire area (≈ 0.5 ha *Jhum* land and 0.1 ha backyard) of the individual farmers and 2 households were selected for the studies. The diversity index was measured as Simpson index, Shannon’s diversity index, and Evenness Index (27–29).

**Table 1:** Popular land races of aroid landraces in the Rombagre village of West Garo Hills of Meghalaya and their ethnobotanical uses.

Sl. No.	Landraces	Description
1	Tamachongkam	Landrace with fused multi corm type Leaves used for pig feeding High yield and market value Cover largest areas under *Jhum* field Tuber are rich in oxalate content (0.82%) and acridity used for curry preparation with meat The acridity is removed by boiling in water before curry preparation.
2	Rengama	A dasheen type landrace, grown widely in *Jhum* land Corms are medium in oxalate content (0.18–0.23%) and acridity, cooked for snacks and curry preparation. High in yield, quality and storage life Rank second in terms of areas under *Jhum* field High incidence of corm borer. There are two type of genotype, i.e., yellowish white and pink budded landraces
3	Tamachok	Bunda type, grown in *Jhum* land for peduncle and corms for cooking Good in taste, low in oxalate content (0.18–0.22%) content and acridity and used for curry preparation. Rank third in terms of area under *Jhum* land
4	Tasakrek	Eddo multicormel type, grown in Jhum field and characterized by number of shoots from single corm. Low in oxalate (0.12%) and acridity, used for baby food. The peduncle and petioles are also used for cooking with fish and meat.
5	Tagiting	*Xanthosoma* spp. There are two variants, i.e., white and purple type. Vigorous in growth habit. Cultivated for the petiole as well as leaves.
6	Ganima	*Colocasia gigantea* a cultivated for petioles, corms are rich in starch (23.87%) and medium in oxalate content (0.28%) content.
7	Chigi	*Colocasia fallax*, grown in backyard nearby drain water under partial shade. Leaves and petioles is used for the cooking during lean period.
8	Other land laces: Pugarkusu Rangdubi Takiltom Tamitim Tadikil Gilasa	These landraces are grown in backyards for the family consumption Rangdubi: Not cultivated widely due to low yield and poor marketing
9	Extinct landraces: Tasupok Tarengsi Tachongchang	Extinct due to smaller size of corm and cormels and poor yield.
10	Giant taro	*Giant taro* (*Alocasia* spp.) grown wild near streams and ponds. The top of big size corms are consumed in some parts of the Tripura.

### Crop evaluation for yield and quality attributes

Total 53 genotypes comprising of 16 landraces from project site (Rombagre, Meghalaya), 18 popular varieties/lines as well as 19 collections form other northeastern states were evaluated at ICAR Research Complex for NEH Region, Umiam, Meghalaya during March – November, 2016–2018 (3 years). The crops were grown following recommended package of practices for the region (30). The experiment was carried out in a randomized block design with three replications. The observations were recorded for growth and yield-related traits such as plant height (cm), petiole length (cm), number of side shoots, average corm and cormel weight (g), yield per plant (kg) and yield (t ha^−1^). The mean values of six plants in each replication were used for the statistical analysis.

### Proximate analysis

Four important quality parameters namely dry matter, total sugar, starch and oxalate content was estimated for all the 53 cultivated genotypes. The dry-matter content in the samples (corms in dasheen type, and cormels in eddo type) were determined by oven-drying 10 g of the sample at 60°C, till a constant weight was obtained (31). Total sugars were estimated by titration, using Fehling’s solution and methylene blue indicator (31). Amount of starch present in the samples was determined using anthrone method (31). After the sugars present in a sample leached out, the starch was hydrolyzed using acid, and estimated as follows:


Starch(%)=ReducingSugar(%)x0.9

Moreover, total oxalate content (dry weight basis) was determined as per CTCRI manual (32).

### Statistical analysis

Mean values of each replication were used for analysis of variance as per Panse and Sukhatme (33). Phenotypic and genotypic variances of the genotypes were estimated as described by Burton and Devane (34), heritability as described by Hanson et al., 1956 (35) and genetic advance was estimated using the formula suggested by Johnson et al., 1955 (36). The genotypic and phenotypic correlation coefficients and path coefficient were estimated as suggested by Dewey and Lu (37). Clustering was performed using the stats package in R 4.2.1 and visualized using the Factoextra package. Principal component analysis was performed using the Factoshiny package in R 4.2.1.

### Molecular characterization

#### Plant materials

Total 58 genotypes including 16 cultivated local landraces collected from the project site (Rombagre, Meghalaya, India) were used for molecular analysis.

#### DNA extraction

The total gDNA was extracted from young leaf tissue by using CTAB method (38) with an addition of Polyvinylpyrrolidone (1%). The sample was then ground to a fine powder using liquid nitrogen. DNA sample concentration was determined using a spectrophotometer (Shimadzu, Jiangsu, China) and they were diluted to 20 ng/μL prior to polymerase chain reaction (PCR) amplification.

#### Molecular analysis

Total 45 SSR markers, reported earlier (22–25, 39) in aroid species were used for the initial screening, 34 shown amplification and except marker Xuqtem-110, remaining 33 were found polymorphic (Table 2). Moreover, markers Ce1B-12, Taro-5, Taro-7, Taro-9, Taro-10, Taro-15, Taro-16, Taro-17, and Taro-18 failed to amplify. Marker Taro-2 and Taro-6 shown amplification in few genotypes only. The PCR analysis was carried out in 20 μL volume containing 40 ng template DNA, 0.5 U TaqDNA polymerase, 0.2 mM each dNTP, 0.2 μM forward and reverse primer each in (1×) reaction buffer that contained 10 mMTris–HCl (pH 8.3), 50 mMKCl, and 2.5 mM MgCl_2_ (Thermo Scientific, Bangalore, India). Amplification conditions (Applied Biosystems Veriti™, Singapore) were initial denaturation at 94°C for 5 min and 35 cycles at 94°C for 60 s and then 50 – 66°C for 60 s, and extension at 72°C for 2 min, followed by 10 min at 72°C and indefinite soak at 4°C. Amplified products were resolved on 3.5% SFR agarose gel containing ethidium bromide (10 mg/mL) at a constant voltage of 80 V for 3 h using a horizontal gel electrophoresis system (Biorad, Singapore). The gel was run in 1 × TBE buffer. A 50 bp DNA ladder (MBI Fermentas) was run alongside the amplified products to determine their approximate band size. Similarly, the amplified products were visualized under UV by image analyses.

**Table 2:** Details of SSR markers used for molecular analysis of aroid species.

Marker	Forward	Reverse	Allele size (bp)	Ann. temp	NA	NE	Obs Hom	Obs Het	Nei	PIC
Ce1A06	GCTTGTCGGATCTATTGT	GGAATCAGTAGCCACATC	95–140	60	6.00	3.41	0.98	0.02	0.71	0.51
Ce1B02	GCACGTTAGACTATTGGA	GTGCTTAGATGGTTGAGA	90–100	60	3.00	2.83	1.00	0.00	0.65	0.36
Ce1B03	TTGCTTGGTGTGAATG	CTAGCTGTGTATGCAGTGT	70–110	50	6.00	3.60	0.83	0.17	0.72	0.61
Ce1B09	AACACTCCCAGAAGAACC	CGTCTTTCAAACTGATCG	50–80	60	5.00	3.72	0.66	0.34	0.73	0.59
Ce1D12	GAAACGTGGGGATTG	CGTTGTGTAAACGGAAG	80–120	56	4.00	3.24	0.79	0.21	0.69	0.51
Ce1F04	AGGGAATACAATGGCTC	ACGAGGGAAGAGTGTAAA	70–90	60	4.00	1.56	0.88	0.12	0.36	0.13
Ce1F12	CTTAGCGTTGTTCCCTAC	GATGCCTGTCCTTATGTTT	50–70	57	6.00	4.88	0.41	0.59	0.79	0.73
Ce1H12	TAGTTAGCGTGCCTTTC	CAACAACTTAATGCTTCAC	50–70	55	4.00	2.63	0.91	0.09	0.62	0.50
AC3	AGTGGCATCAATGGAGGA	CCACTAAACGACGACCCAC	120–200	54	5.00	3.64	0.98	0.02	0.73	0.60
HK5	CCCACCTCTTCCCATTCGCTT	CGATCCTTCCAGCTCCGACAT	160–260	55	7.00	5.10	0.33	0.67	0.80	0.76
HK7	GTTGTCCGCCTGTGCGTTCT	CTCTTGGGAATTCTCCGGGTG	125–165	56	6.00	3.32	0.74	0.26	0.70	0.61
HK22	ACATCAAACCTCTGGTGGGC	AGCAATCCTAGCCGAGGTG	150–300	53	7.00	3.25	0.33	0.67	0.69	0.62
HK25	TGACTAGGCAGGAAGGTAA	CAAGCATTCTCTGAACTATG	120–200	51	5.00	3.85	0.50	0.50	0.74	0.67
HK26	GGGTGTTATCGCCATAGTCAT	GAAACACCACAACGGAGAAAC	110–175	51	7.00	4.04	0.53	0.47	0.75	0.70
HK29	GTCTGTGGAACCCTCAAGC	ATTGTGGGAGCGATAGGG	130–210	54	6.00	3.48	0.88	0.12	0.71	0.62
HK31	TACCGCCGAGTGCTTATC	TACGGCTGGAATCAAAGC	140–220	51	5.00	1.83	0.59	0.41	0.45	0.37
HK34	TTACTCCAAACGAGGCAAAC	CCTTCAAGATGTTACCAAATGC	180–290	56	8.00	5.09	0.16	0.84	0.80	0.74
HK-35	TACTAGAACCCCGTCAGTCT	CGTCGATTTATCAGTGAGC	240–300	53	5.00	3.83	0.52	0.48	0.74	0.66
HK38	AAACGCGGCCAGAAGATC	GAATAGCGGAACAAGGTAGA	120–190	54	5.00	2.82	0.98	0.02	0.65	0.54
Taro01	CTGACTCTTGTAAGGTCGCTC	CAAAAGCAGGTCTGGATG	100–130	56	5.00	4.36	1.00	0.00	0.77	0.65
Taro03	CGTGAGGGCGGTTTTGTCAGG	ACGAGCGAGCAGCTCACCGC	200–210	60	3.00	2.94	1.00	0.00	0.66	0.37
Taro04	ACTTTATGTAATAGTGAACATT	CGAAGCAGCGCCACCGGC	420–450	57	4.00	3.74	0.98	0.02	0.73	0.59
Taro11	CGGCCAAGAAGGAGAGCCA	ACAAGCTTATTTATAGTGGCTA	300–550	58	4.00	2.85	1.00	0.00	0.65	0.45
Taro12	CGCTTTGCCTTTCGGTGTTGAGA	ACTTGGTGTGCAGCAAGACTT	710–810	56	4.00	3.36	0.97	0.03	0.70	0.52
Taro13	GTTAATGGGATATAAACGGCA	CGCCAAAGTCTATTGAGTGTT	620–650	58	4.00	2.29	1.00	0.00	0.56	0.32
Taro14	ACAAAATATGTTCTCTGTGATAT	ACCTAGTCTACTATCGAGCCA	590–660	55	6.00	5.55	0.21	0.79	0.82	0.74
Taro19	TTCGACGTACCGATCGAGACCG	TTACCGAGACTGACGAAGCTAG	380–425	54	4.00	2.59	1.00	0.00	0.61	0.40
Xuqtem-84	AGGACAAAATAGCATCAGCAC	CCCATTGGAGAGATAGAGAGAC	325–500	65	7.00	4.79	0.88	0.12	0.79	0.69
Xuqtem-110	AGCCACGACACTCAACTATC	GCCCAGTATATCTTGCATCTCC	510–600	66	4.00	3.55	1.00	0.00	0.72	0.54
Xuqtem-73	ATGCCAATGGAGGATGGCAG	CGTCTAGCTTAGGACAACATGC	310–330	66	3.00	1.94	0.81	0.19	0.48	0.23
Xuqtem-55	CTTTTGTGACATTTGTGGAGC	CAATAATGGTGGTGGAAGTGG	200–220	60	5.00	4.86	0.31	0.69	0.79	0.69
Xuqtem-88	CACACATACCCACATACACG	CCAGGCTCTAATGATGATGATG	120–220	65	6.00	4.93	1.00	0.00	0.80	0.72
Xuqtem-91	GTCCAGTGTAGAGAAAAACCAG	CACAACCAAACATACGGAAAC	500–520	65	4.00	3.00	1.00	0.00	0.67	0.41
Mean			–		5.06	3.54	0.76	0.24	0.69	0.55

Ann, Annealing temp (°C); NA, Observed number of alleles; NE, Effective number of alleles; Obs Hom, Observed homozygosity; Obs Het, Observed heterozygosity; Nei, Nei’s expected heterozygosity; PIC, Polymorphism information content.

### Data analysis

Only consistent, bright, reproducible (i.e., band absence was randomly verified) SSR bands were scored as present (1) or absent (0), where each character state was treated independently. The summary statistics of SSR markers such as the number of alleles per locus, allele frequency, heterozygosity and polymorphic information index (PIC) were determined using Power Marker version 3.25 (40). Genetic diversity was assessed using both model-based approach and distance based approach. For distance based approach, the unrooted phylogenetic tree was constructed based on genetic distance as per Nei distance (41). Clustering using model based approach was performed using Structure 2.3.4 software with 50,000 burning period length followed by 50,000 Markov Chain Monte Carlo (MCMC) replication (42) with a K value ranging from 1–10. The optimum K value was determined based on delta K value using a web program namely Structure Harvester.^1^

## Results

### Population diversity and ethnobotanical uses

**Figure 2:**
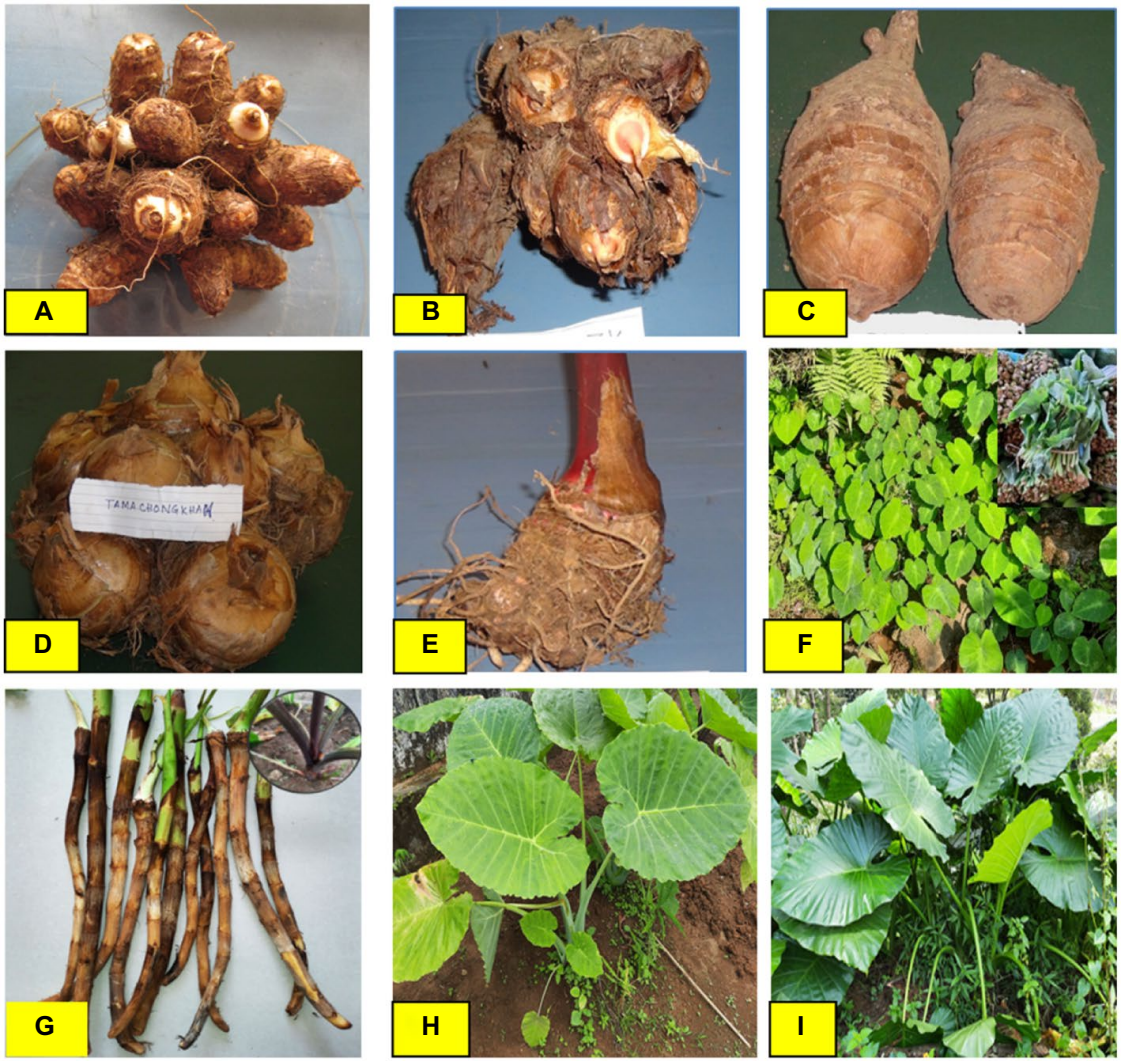
Diverse group of Aroid genotypes in Garo Hills of Meghalaya. **(A)** Eddo (cv. Sunajuli). **(B)** Eddo with fused multi cormels (*cv*. Tasakrek-1). **(C)** Dasheen (*cv*. Rengama). **(D)** buda type with fused multi-corm (*cv*. Tamachongkham). **(E)** *Xanthosoma* spp. (*cv*. Tagiting Purple). **(F)** *Colocasia fallax* (leafy type *cv*. Chigi). **(G)** *Colocasia esculenta* var. *aquatilis* (*cv.* Tharsing). **(H)** *Colocasia gigantea* (*cv*. Ganima). **(I)** Wild Giant taro (*Alocasia* spp.)

**Figure 3:**
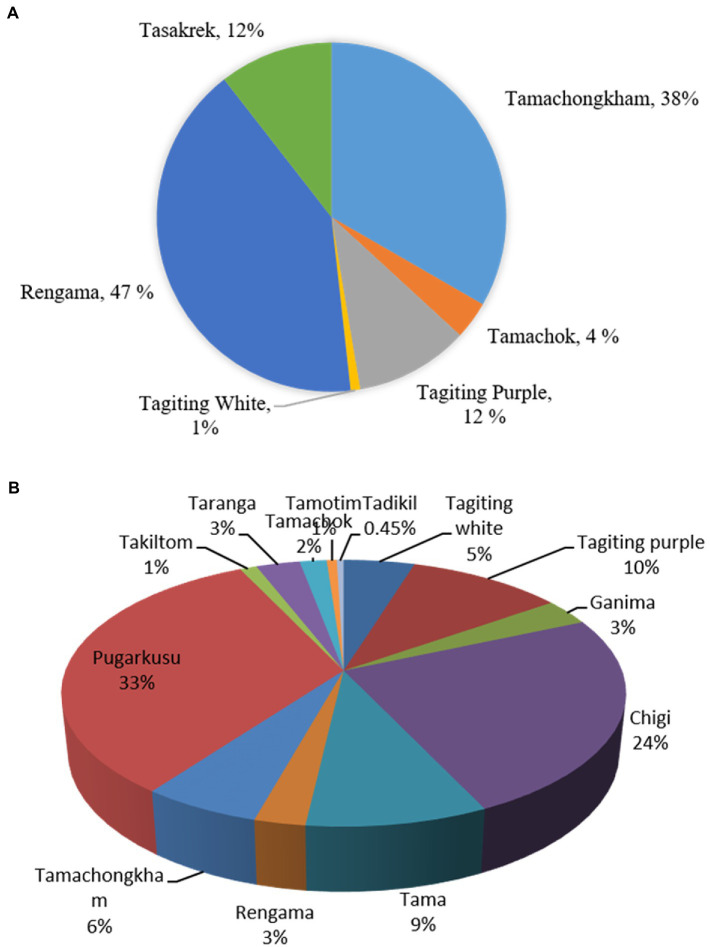
Population diversity of the taro landraces under **(A)**. Jhum field, **(B)**. backyards in Rombagre village of West Garo Hills, Meghalaya (India).

The household population diversity of aroids was assessed at the project site under backyard and *Jhum* farming systems in Rombagre village of West Garo Hills in Meghalaya. A total of sixteen diverse genotypes of aroids were found at household levels including *Xanthosoma* spp., *Colocasia esculenta* var. *aquatilis* (cv. Tharsing), *Colocasia fallax* (*cv*. Chigi), and *Colocasia gigantea* (*cv*. Ganima). These genotypes were of a diverse group with unique ethnobotanical uses (Table 1; Figure 2). Out of 16 genotypes, 6 landraces were grown under *Jhum* land as well as in backyards, while a total of 13 landraces were grown only under the backyards conditions. Based on the population study in *Jhum* land, it was found that landrace Rengama was most dominant in area with a share of 47% of the total population, followed by Tamachonkham (36%), and Tasakrek (12%). However, the maximum area in backyards was under Pugarkusu (33%) followed by *Colocasia fallax* cv. Chigi (24%). The percent populations of the other genotypes are presented in the pie diagram (Figure 3). All the genotypes grown at household level were different from each other for agro-morphological traits and ethnobotanical uses (Table 1). The Simpson’s diversity index value was higher for the backyard (0.80) as compared to *Jhum* field (0.63). Similarly, the Shannon diversity index was also higher (42.59) in the backyards as compared to *Jhum* field (17.81). Likewise, the evenness index (EI) was 0.42 for the *Jhum* field, and 1.0 for the backyards. Besides, some of the genotypes differ in agro-morphological traits like, Rengama with cream and pink bud eyes, and Tasakrek with a difference in the size of the corm and cormels but known by a common local name.

### Estimation of genetic variation

The analysis of genetic parameters has shown the presence of significant variation (*p* < 0.05) among the genotypes of the aroid species for all the yield and quality attributes (Table 3). Yield attributing traits like, plant height range from 89.4–206.1 cm, petiole length (61.4–145.08 cm), number of side shoots (1.84–5.92), corm weight (38.0–683.3 g), cormel weight (14.87–348.3 g), and yield per plant (0.24–1.83 kg). Among the genotypes, maximum plant height (206.1 cm) and petiole length (145.08 cm) were observed in Rengama-1, a dasheen type genotype collected from the Garo Hills of Meghalaya, while the maximum number of side shoots (>5.0) was recorded from commercial cultivars, namely, BCC-1, BCC-1A, Muktakeshi, Sel C-1 and ML-2. Further, the highest corm weight was recorded in dasheen type genotypes C-3 (683.3 g), followed by Panchmukhi (633.3 g), Arcol-7(600 g) and Selection C-3 (550.0 g). Further, the cormel weight was the highest in dasheen type genotypes Rengama-2 (348.3 g), and Rongrem (270.0 g). There were lateral stolons (5–7 per plant) in place of cormels in genotype Tharsing (*Colocasia esculenta* var. *aquatilis*). The average length, diameter, weight and yield of the stolon were 44.8 cm, 1.8 cm, 86.66 g and 476.66 g, respectively. Tamachongkham (31.4 t ha^−1^), Tama (30.15 t ha^−1^), Rengama-2 (28.10 t ha^−1^), Rongrem (28.10 t ha^−1^) and Rengama (27.0 t ha^−1^) were identified as high yielding genotypes under dasheen types. While, White Gauriya (27.53 t ha^−1^) and SJ-1 (25.19 t ha^−1^) were identified under eddo types.

**Table 3:** Estimation of genetic parameters for growth, yield, and quality attributes in Aroid species.

Traits/genetic parameters	Plant height (cm)	Petiole length (cm)	No. of side shoots	Average corm wt. (g)	Average cormel wt. (g)	Yield/plant (kg)	Total sugars (%)	Starch (%)	Oxalate (%)	Dry matter (%)
Mean	118.65	89.35	3.71	249.21	48.14	0.93	3.26	21.65	0.20	21.68
Minimum	89.35	61.47	1.84	38.04	14.87	0.24	1.93	15.34	0.10	16.75
Maximum	206.04	145.08	5.92	683.33	348.33	1.83	4.95	32.49	0.82	27.08
SE(m)	2.33	1.52	0.27	20.15	2.29	0.05	0.09	0.45	0.04	0.23
SE(d)	3.29	2.15	0.39	28.50	3.24	0.01	0.12	0.64	0.06	0.32
CV	3.40	2.94	12.80	14.01	8.24	8.24	4.58	3.62	35.64	1.81
GCV	16.46	22.14	24.54	61.26	116.90	39.26	22.63	16.14	32.72	11.78
PCV	16.80	22.33	27.67	62.84	117.19	40.11	23.09	16.54	48.39	11.92
h2	95.91	98.27	78.61	95.03	99.51	95.78	96.06	95.20	45.73	97.70
GA	39.39	40.39	1.66	306.61	115.63	0.75	1.49	7.02	0.09	5.20
GAM	33.20	45.21	44.81	123.03	240.22	79.01	45.70	32.45	45.59	23.98

Similarly, quality traits including total sugar range from 1.93–4.94%, starch content 15.32–32.49%, total oxalate 0.10–0.82%, and dry matter 16.75–27.08%. Among the genotypes, the highest sugar content was recorded in Arcol-2 (4.94%) followed by Nainital (4.90%). Moreover, the highest starch content was observed in the popular cultivar Kandha-5 (32.49%) followed by White Gauriya (30.32%). The starch content in *Xanthosoma* spp. ranged from 17.67 (Tajiting Purple) to 20.65% (Tagiting White), while in *Colocasia esculenta* var. *aquatilis* (Tharsing) it was 20.23%. Further, among the genotypes, the lowest total oxalate was recorded in SJ-1(0.10%) followed by BK Coll-2 and ML-2 (0.11% each), while the maximum total oxalate was observed in landrace Tamachongkham (0.82%) followed by Tama (0.42) and Panchmukhi (0.38%). The total oxalate content in *Xanthosoma* spp. was about 0.24%. While, in *Colocasia esculenta* var. *aquatilis* (cv. Tharsing), the average starch and total oxalate content were 0.17 and 19.0%, respectively. Further, dry matter content was highest (27.08%) in KCA-1 and Panchmukhi.

The phenotypic coefficients of variation (PCV) were higher than their corresponding values of genotypic coefficients of variation (GCV) for all the characters. The genotypic coefficient of variation contributed significantly to the phenotypic variation for all the traits. All the traits except plant height, starch, and dry matter content have shown the highest GCV and PCV (>20%) indicating higher variability for these traits in the population. Moreover, except total oxalate content, other traits have shown high heritability (> 60%). Further, all the traits have also shown higher genetic advance (> 40%), except for plant height, dry matter, and starch content.

### Principal component analysis

**Figure 4:**
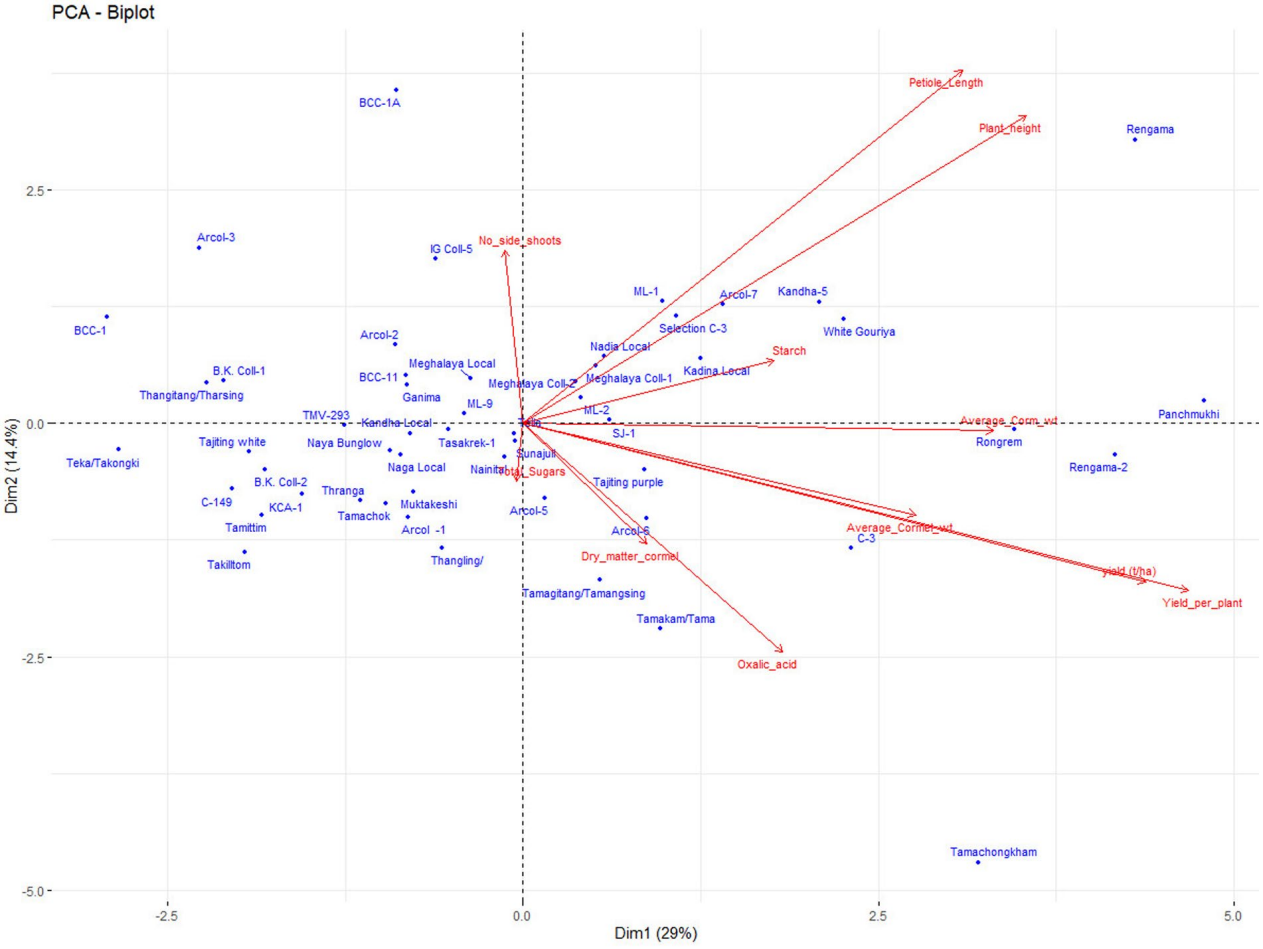
Principal components analysis biplot for traits and genotypes.

The results of PCA analysis also revealed the presence of variability for different traits (Figure 4). The first four components with an Eigenvalue of >1, and contributed 65.97% of the total variation. Principal component 1(PC1), contributed 29.0% of the total variability (with loading >3.19), which was positively attributed by growth and yield attributing traits such as plant height, petiole length, yield per plant, yield per ha, corm and cormels weight. PC2 contributed 14.4% to the total variability and was depicted mainly by plant height, petiole length, and the number of side shoots. The PC3 contributed 12.19% of the total variability and was mainly attributed to quality traits such as total sugar, dry matter, and starch content. PC4 contributed 10.36% to the number of side shoots, cormel weight, and starch content. The PCA biplot analysis also differentiated the genotypes for the different traits like Tamachongkham and Tama for higher oxalate content, Rengama for plant height and petiole length, C-3, Panchmukhi, Rengama-2, and Rongrem for corm weight and total yield (Figure 4).

### Association between yield and quality attributes

The genotypic correlation coefficients for yield and quality contributing characters were higher than phenotypic correlation coefficients in most cases (Table 4). For traits, like the number of side shoots, total sugar, total oxalate, starch, and dry matter contents, phenotypic correlation coefficients were the same as or higher than the genotypic correlation coefficients. Among the traits at the genotypic and phenotypic level, yield per plant was significant and positively correlated with plant height, petiole length, number of side shoots, corm, and cormel weight (Table 4). However, among the quality parameters the sugar content was negatively correlated with all the traits except for corm weight. Further, starch content was significant and positively correlated with yield, corm weight, petiole length, and plant height. Moreover, total oxalate content was negatively correlated with the number of side shoots, and total sugar content at both genotypic and phenotypic levels. Likewise, dry matter content was significant and positively correlated with quality traits like, total sugar and starch content but negatively correlated with total oxalate, as also indicated by phenotypic correlation.

**Table 4:** Genotypic (G) and phenotypic (P) correlation for growth, yield and quality attributes in Aroid spp.

Traits		Petiole length (cm)	No. of side shoots	Average corm wt. (g)	Average cormel wt. (g)	Yield/plant (kg)	Total sugars (%)	Starch (%)	Oxalate (%)	Dry matter (%)
Plant height (cm)	G	0.873^**^	0.024	0.405^**^	0.234*	0.498^**^	−0.082	0.170^*^	0.119	0.015
	P	0.837^**^	0.027	0.384^**^	0.232*	0.478^**^	−0.082	0.159^*^	0.122	0.019
Petiole length (cm)	G		0.114	0.245^**^	0.209^*^	0.417^**^	−0.063	0.167^*^	0.078	−0.058
	P		0.089	0.240^**^	0.207^*^	0.410^**^	−0.063	0.167^*^	0.077	−0.059
No. of side shoots	G			−0.175	0.203^*^	0.049	−0.043	0.065	−0.349^**^	0.149
	P			−0.145	0.178^*^	0.055	−0.013	0.052	−0.279^**^	0.129
Average corm wt. (g)	G				0.238^**^	0.538^**^	0.141	0.178^*^	0.027	0.290^**^
	P				0.231^**^	0.523^**^	0.137	0.178^*^	0.023	0.273^**^
Average cormel wt. (g)	G					0.485^**^	−0.002	0.098	0.106	0.043
	P					0.477^**^	−0.002	0.094	0.101	0.043
Yield/plant (kg)	G						−0.030	0.287^**^	−0.104	0.184^*^
	P						−0.021	0.285^**^	−0.101	0.178^*^
Total sugars (%)	G							0.042	−0.183^*^	0.180^*^
	P							0.041	−0.173^*^	0.180^*^
Starch (%)	G								−0.033	−0.005
	P								−0.035	−0.002
Oxalate (%)	G									−0.172^*^
	P									−0.168^*^

*, ** significant at *p* < 0.05 and *p* < 0.01, respectively.

### Genetic diversity

#### Genetic diversity based on quantitative traits

Based on 10 yield and quality related attributes, all 53 genotypes were grouped into 4 major clusters (Table 5). Cluster-I was comprised of 7 genotypes and characterized by small size corms (<150 g) and cormels (< 25 g). Cluster-II was the largest cluster with 24 genotypes including Ganima (*Colocasia gigantea*) having medium size corms (150–250 g) and cormels (25–50 g) which was further grouped into two sub groups, i.e., Cluster II-A (13 genotypes), and cluster II-B (11 genotypes including Tajiting White a *Xanthosoma* spp.). Cluster III was comprised of 6 high yielding (1.53 kg/plant) dasheen type genotypes like Tamachokgkham, Rengama-2, Rongrem, and Rengama collections from Garo hills and popular varieties/lines like Panchmukhi and C-3 with higher plant height, petiole length, maximum yield per plant, and rich in dry matter content. Furthermore, cluster-IV comprised 16 genotypes, including Tagiting Purple (*Xanthosoma* spp.) having the maximum number of side shoots. It was further grouped into two subgroups, i.e., IV-A having prominent corm (>400 g) with medium size cormels (25–50 g) and IV-B with medium size corm (>150 g) and cormels (25–50 g) with low total oxalate and higher in starch content.

**Table 5:** Cluster mean values for different yield and quality attributes.

Cluster	Plant height (cm)	Petiole length (cm)	No. of side shoots	Average corm wt. (g)	Average cormel wt. (g)	Yield /plant (kg)	Total sugars (%)	Starch (%)	Oxalate (%)	Dry matter (%)
Cluster-I	114.92	89.52	4.11	130.01	17.97	0.341	2.93	21.11	0.20	20.48
Cluster-II-A	113.19	88.11	3.58	182.44	36.74	0.921	3.40	20.24	0.19	21.50
Cluster-II-B	111.50	77.53	3.27	228.16	29.82	0.656	3.45	20.97	0.20	21.95
Cluster-III	146.49	107.37	3.43	464.63	146.06	1.537	3.44	22.43	0.23	22.85
Cluster-IV-A	123.47	97.73	4.61	510.22	49.73	1.110	3.45	23.58	0.27	21.57
Cluster-IV-B	117.26	88.88	3.78	188.89	44.98	1.148	2.92	23.02	0.17	21.79

#### Genetic diversity based on molecular markers

**Figure 5:**
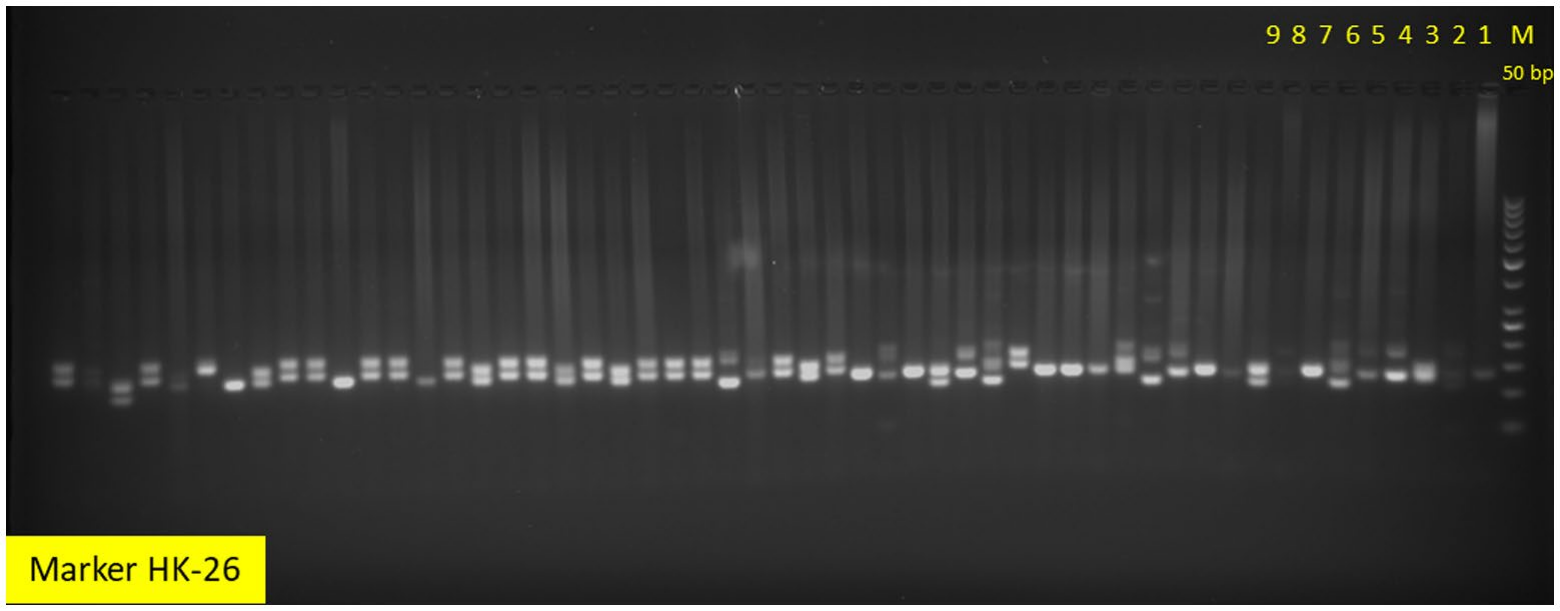
Allelic variations among aroid genotypes by SSR marker HK-26.

The SSR markers (33) used in the study has shown wider allelic variations among the genotypes (Table 2; Figure 5). A total of 136 alleles were observed with the minimum (3) in markers Ce1B-02, Taro-03, Taro-13, and Xuqtem-73 each and a maximum (8) in HK-34. Likewise, the effective number of alleles varied from 1.56 (Ce1F-04) to 5.10 (HK-5). The population has also shown the presence of heterozygosity, and it ranges varied 0.00–0.84. The observed average heterozygosity (0.24) was less than expected heterozygosity (0.69). Among the groups, the maximum heterozygosity was observed in eddo and dasheen groups (0.66 each) followed by dasheen fused type (0.56), eddo fused type (0.51), *Xanthosoma* spp. (0.27), swamp taro (0.06), and the least in eddo leafy types (0.09). Further, the polymorphic information content (PIC) value ranged from 0.13 to 0.76 across these 56 genotypes with a mean PIC value of 0.55. Out of 33 SSR markers 24 markers showed PIC value ≥0.50.

**Figure 6:**
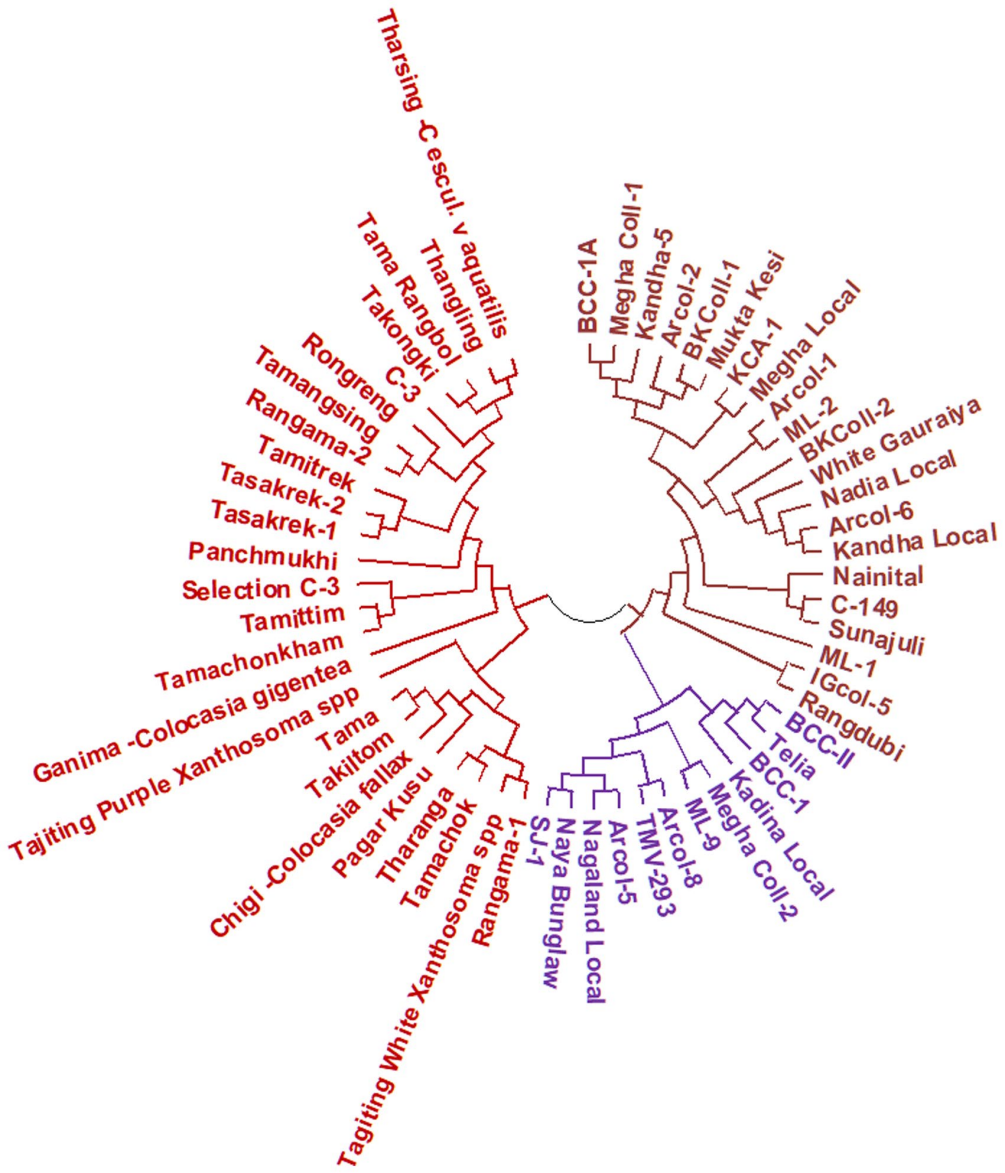
Dendogram depicting genetic relationship among the Aroid genotypes based on SSR markers.

The results of molecular variance analysis (AMOVA) showed that the presence of diversity within and between the groups of the genotypes (Table 6). The maximum variation was observed within populations (57.11%) followed by between individuals (32.46%) of the same population and among populations (10.43%). Further cluster analysis has also shown wider diversity among the genotypes of aroid species (Figure 6). All the genotypes were grouped into 3 major groups and Cluster-I (21 genotypes) and II (12 genotypes) were comprised of popular cultivars and landraces collected from the other parts of the country while the landraces of Garo Hills including related species *Colocasia gigantea* (cv. Ganima), *Colocasia esculenta* var. *aquatilis* (*cv*. Tharsing), *Xanthosoma* spp. (Tajiting Purple and Tagiting White) grouped in Cluster-III (25 genotypes). Tharsing a stolon-type taro was found to be most diverse from rest of the species. Moreover, *Colocasia fallax cv*. Chigi a leafy type accession was found closer to the local landrace Takiltom and Tama.

**Table 6:** AMOVA design and results (average over 33 loci).

Source of variation	Degree of Freedom	Sum of squares	Variance components	Percentage variation
Among populations	6	190.98	1.26	10.43
Among individuals within populations	51	904.07	6.90	57.11
Within individuals	58	227.50	3.92	32.46
Total	115	1322.55	12.08	

**Figure 7:**
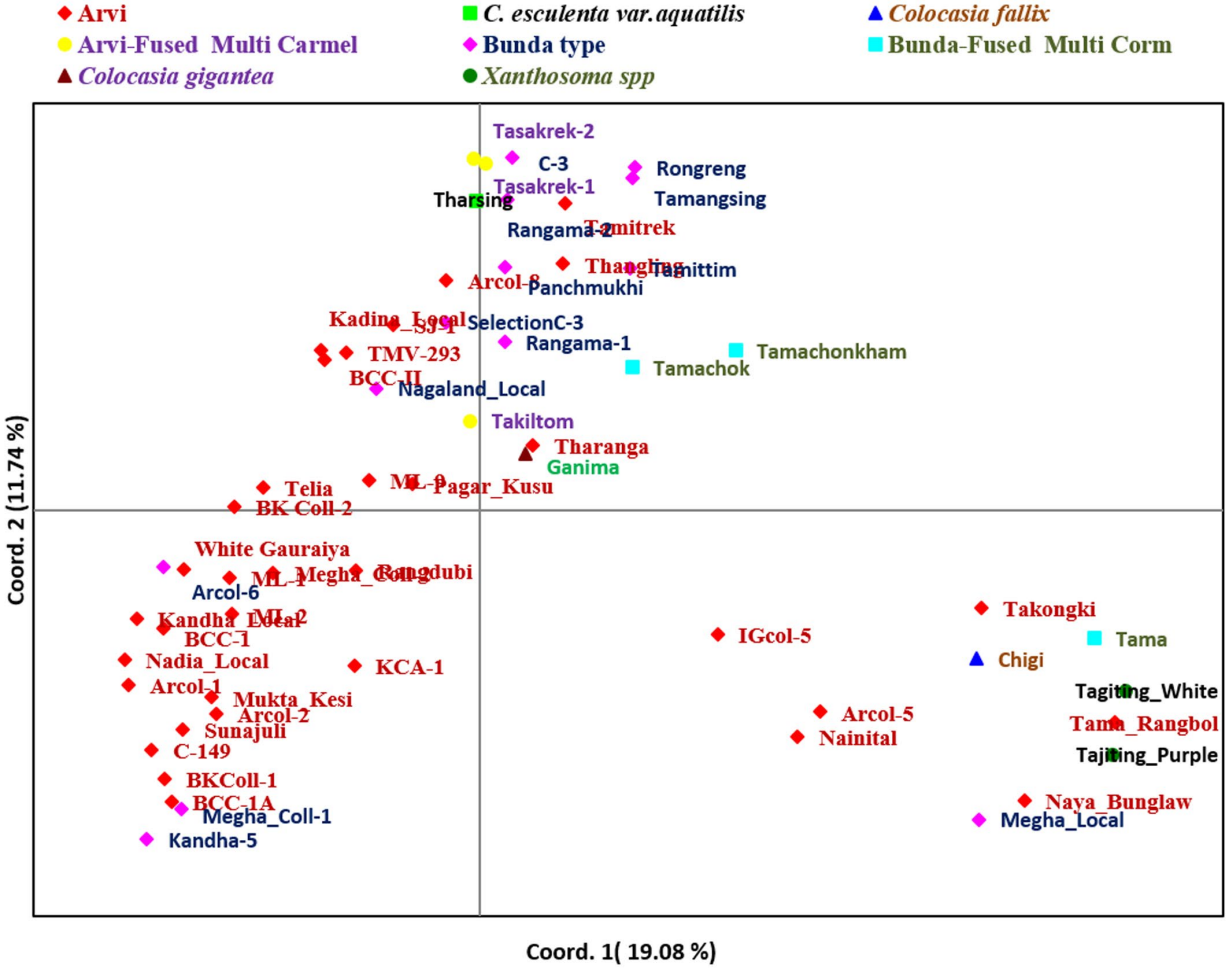
Principal coordinate analysis of Aroid genotypes based on SSR markers.

Further, principal coordinates analysis (PCoA) revealed that the three most important principal coordinate axes accounted for 36.84% of the total variance (Figure 7). The first coordinate axes differentiated the local landraces with popular cultivars. The second axes also differentiated the related aroid species (*Xanthosoma* spp.,) and *Colocasia fallax* (*cv*. Chigi) to *Colocasia esculenta* and species *Colocasia gigantea* (*cv*. Ganima), *Colocasia esculenta* var. *aquatilis* (*cv.* Tharsing) and genotypes having multi-corm, multi-cormels with other popular cultivars of *Colocasia* spp.

#### Genetic structure and interrelationship

**Figure 8:**
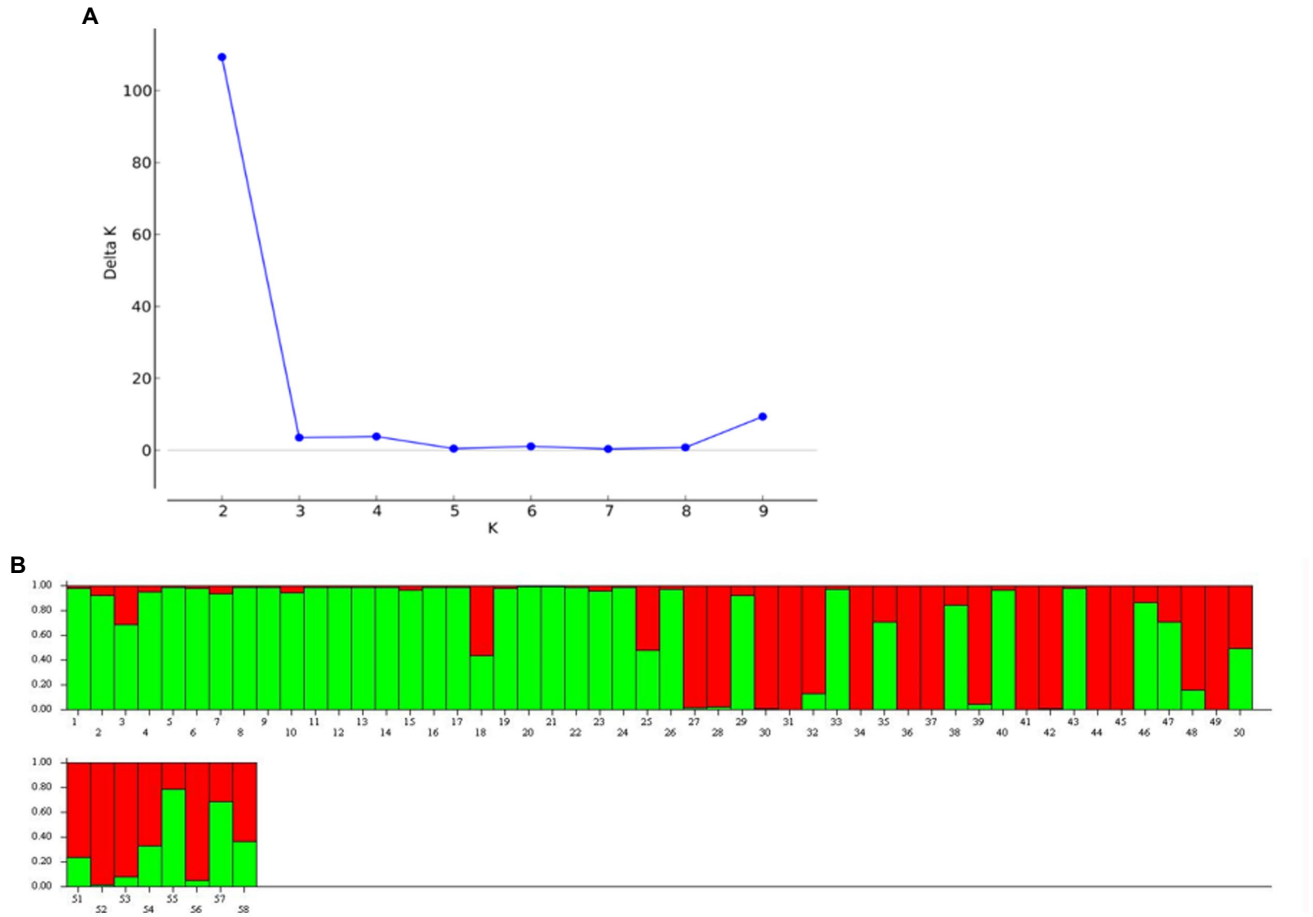
Population structure of 58 Aroid genotypes based on 33 SSR markers. **(A)** ∆ K graph, **(B)** population structure at ∆K = 2.

**Figure 9:**
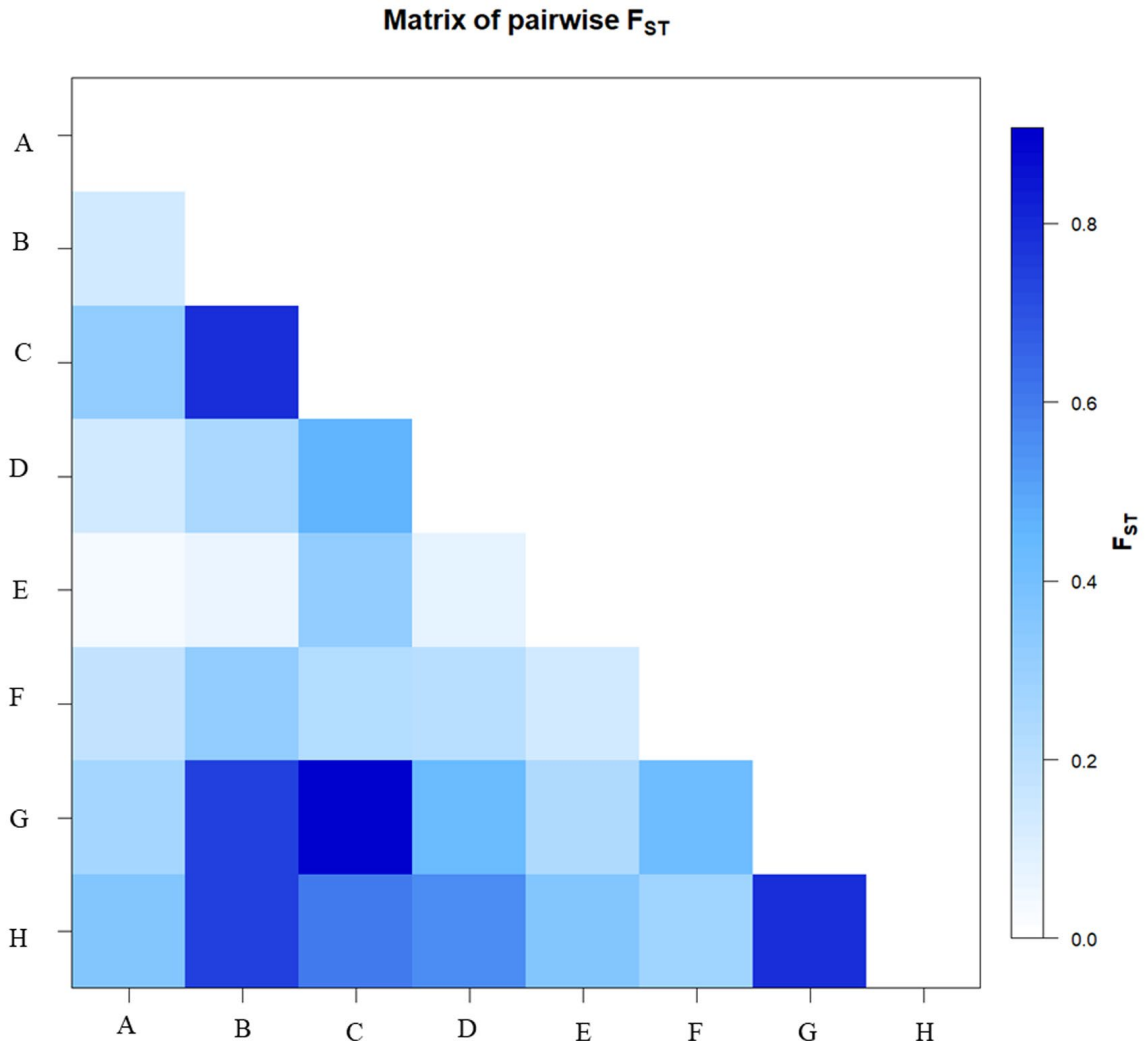
Matrix of pairwise F_ST_. Where: A: Eddo; B: *Colocasia esculenta* var. *aquatilis* (cv. *Tharsing*); C: *Colocasia fallax* (cv. Chigi); D: Eddo with fused multi-cormel; E: Dasheen type; F: Dasheen type with fused multi-corm; G: *Colocasia gigantea* (cv. Ganima); H: *Xanthosoma* spp.

Genetic structure analysis was carried out based on the 33 microsatellite markers as per the procedure described by Evanno et al., 2005 (43). The analysis has detected the maximal ΔK (109.33) at *K* = 2. The Δ K value decreases with a further increase in K (Figure 8). Out of 58 genotypes, 34 were admixture, and it was more common among the popular cultivars and landraces collected from other parts of the country (Figure 8). Moreover, there was the least and no admixture was observed in other aroid species *Xanthosoma* spp. (1 & 8), leafy type *Colocasia fallax* (2), stolon type *Colocasia esculenta* var. *aquatilis* (16) and *Colocasia gigantea* (23). The genetic differentiation between the groups was also from low to very high with fixation index (F*_ST_*) values of 0.03–0.80 (mean = 0.28). The maximum differentiation was observed between *Colocasia fallax* (cv. Chigi) to *Colocasia esculenta* var. *aquatilis* (*cv*. Tharsing); *Colocasia fallax* (*cv*. Chigi) to *Colocasia gigantea* (*cv*. Ganima) and *Colocasia gigantea* (*cv*. Ganima) to *Xanthosoma* spp. (Figure 9). Moreover, the least differentiation was observed between Eddo/Arvi (*Colocasia esculenta* var. *antiquorum*) and bunda/dasheen type (*Colocasia esculenta* var. *esculenta*) genotypes.

## Discussion

Aroid species are a group of multipurpose tuber crops, grown widely in tropical and subtropical parts of the world for their tubers, petioles and leaves, and playing an important role in the nutritional security of the population. The present study has indicated the presence of diverse genotypes (16 nos.) of aroid species with distinct ethnobotanical uses at household levels in the backyards and *Jhum* land in West Garo Hills of Meghalaya. The maximum diversity was observed in the backyards over the *Jhum* field. The Simpson’s and Shannon’s diversity index values were 0.80 and 42.59, respectively, for backyards and *Jhum* field, indicating the presence of higher diversity in the backyards as compared to the *Jhum* field. Moreover, the low evenness index value (0.42) for the *Jhum* field as compared to the backyard (1.0) indicated the dominance of some genotypes in the backyards as compared to the *Jhum* field. Further, evaluation trials have also shown wider variability for growth, yield, and quality attributes in aroid genotypes collected from the region. Angami et al., 2015 (44) also observed wider variability in taro landraces of the northeastern India.

Although, the crop is propagated clonally using corms, cormels, and shoots, there is scope for crop improvement through the selection of superior lines, as indicated by both the genotypic and the phenotypic coefficient of variation for all the traits (Table 3). All the traits, except, plant height, starch and dry matter content has shown the highest GCV and PCV (> 20%) indicating higher variability for these traits in the population. Except for total oxalate content, other traits have shown high heritability (> 60%). Further, except for plant height and dry matter content all the traits have shown a higher genetic advance (> 40%). Similar findings were also observed by Singh et al., 2003 (45) in taro. The high heritability and genetic advance of traits, suggest the least effect of environment and possibly the prevalence of additive gene action. Such characters would be responsive to selection (34, 36). The traits like plant height, starch, and dry matter content shown high heritability and low or moderate genetic advance indicating expression of these traits are influenced by non-additive gene action and environment. Further, PCA analysis also revealed the presence of variability for different traits. Economically important traits like yield, corm weight, dry matter, total sugar, and total oxalate content, as explained by principal components, contributing 65.9% of the total variation and selection can be followed for these traits in the population. PCA biplot also differentiated the genotypes for most of the traits, and hence the superior genotypes from PC-I can be selected for specific traits (Figure 4).

Further, cluster analysis based on quantitative traits has also revealed the presence of wider diversity among the groups (Table 5). The identified high yielding (> 25.0 t ha^−1^) landraces Tamachongkham, Tama, Rengama-2, Rongrem and Rengama collections from Garo Hills (all dasheen type) and eddo type genotypes White Gauriya and SJ-1, of cluster –III can be promoted for commercial production in the region. These genotypes outperformed the recommended cultivar Muktakeshi for yield by 11.71–29.18%. As yield-attributing traits like, corms and cormels weight was found highly heritable, the genotypes can be selected form the respective groups as per the choice of the consumer and market. For example, a genotype with smaller corm and cormels from Cluster-I, a prominent corm with small cormels from Cluster IV-A and a genotype with medium size corm and cormels from the cluster IV-B. Moreover, the collection of aroids from the project site (Rombagre, Garo Hills) have also shown wider variability for different traits (Table 1).

Under ethnobotanical uses, genotypes like Tamachongkham, Tama with high acridity and total oxalate content was used only for cooking with meat (Supplementary Table S1). For curry preparation, they use genotypes like Rongrem, Tamachok etc. which are low to medium in acridity and total oxalate (< 0.25%) content. Tasakrek, a landrace with low in acridity, total oxalate content (0.10–0.12%) and high in total sugar content (> 3.0%) was utilized as baby food. As breakfast snacks, landraces like Rengama and Tamittim having higher level of starch (>22%), and lower level of acridity and total oxalate (< 0. 20%) were used. Tania (*Xanthosoma* spp.) landraces such as, Tagiting Purple and Tagiting White grew vigorously and were grown mostly for their petioles for cooking and pig feed especially during dry winter season, when there is scarcity of vegetables. This could be due to prolonged periods of availability, tolerance to leaf blight, and low temperature as compared to other *Colocasia* species, as *Xanthosoma* spp. are rich in cuticular wax content (46). Moreover, tania has been found rich in phosphorous in leaves (388 mg/100 g), petioles (80 mg/100 g), and β-carotene (3,300 ug/100 g) content in leaves (47). Further, the landrace Chigi (*Colocasia fallax*) is grown in the backyards for its leaves and petioles as green leafy vegetables during winter and spring–summer when there is a scarcity of vegetables in the villages. It has been found tolerant to shade and excessive moisture, as it is grown near the drains having multiple fruit plants (banana, orange, jackfruit, guava etc.). Ganima (*Colocasia gigantea*) corms are rich in starch (23.87%) and medium in total oxalate (0.28%) content. Its vigorous growth habit/higher petioles yield with low acridity and poor tuber yield (8.3 t ha^−1^) may be the possible reasons for petioles production. At household levels, the loss of genetic diversity was also observed, and which was mainly due to factors like poor yield, poor marketing and being susceptible to pests and diseases etc. (Table 1).

The genotypic correlation coefficients for yield and quality contributing characters were higher than the phenotypic correlation coefficients in most cases (Table 4), indicating that the effects of environment has suppressed the phenotypic relationship between these characters. Higher genotypic than phenotypic correlation coefficients among the various traits have been reported by Paul et al., 2014 (48) and Mukherjee et al., 2016 (49) in taro. For traits like the number of side shoots, total sugar, total oxalate, starch, and dry matter contents, phenotypic correlation coefficients were the same as or higher than the genotypic correlation coefficients, indicating that both environmental and genotypic correlations in these cases acted in the same direction and finally maximized their expression at the phenotypic level.

Among the traits at the genotypic and phenotypic levels, yield per plant was significant and positively correlated with plant height, petiole length, number of side shoots, and average corm and cormel weight (Table 4). Similar findings were also reported by Paul et al., 2014 (48). However, among the quality parameters, the total sugar content was negatively correlated with all the traits except for corm weight. Further, starch content was significant and positively correlated with yield, corm weight, petiole length, and plant height. Moreover, total oxalate content was negatively correlated with the number of side shoots and total sugar content at both genotypic and phenotypic level. Likewise, dry matter content was significant and positively correlated with quality traits like, sugar and starch content but negatively correlated with total oxalate. Similar is the case with phenotypic correlation. This could be due to the higher accumulation of photosynthates by the plants and translocation towards corm and cormel production. Moreover, selection based on higher dry matter, starch, and sugar content will be effective to identify the superior accession low in acridity and total oxalate content.

The results of molecular analysis (33 SSR), have generated a total of 136 alleles with wider allelic variations among the genotypes and ranges from 3 (Ce1B-02, Taro-03, Taro-13 and Xuqtem-73) to 8 (HK-34). The presence of heterozygosity (0.0–0.84), indicated the natural hybridization and evolution of the different landraces. Further, group wise maximum heterozygosity in eddo and dasheen also indicated the natural hybridization among the accessions of the same group. Moreover, the least heterozygosity in eddo leafy type and Swamp taro may be due to difference in ecological habitat and a lack of natural crossing. Among the markers (33), the PIC values ranges from 0.13–0.74 and majority of the markers (24) showed high polymorphism (PIC >0.5), and thus indicated the presences of a higher level of genetic diversity among the genotypes which markers can easily differentiate. Similarly, higher polymorphism has also been observed by Khatemenla et al., 2019 (50) in the genotypes of northeastern India. Such greater diversity is expected, as the region is considered the primary center of diversity of the Aroid species. Further, marker HK-34 differentiated Chigi, Tamachok, Tasakrek-1, Taskrek-2, Megha Local, and Rengama having multiple copies (3) of the alleles from other genotypes with unique alleles at an extra locus. This could be due to differences in ploidy levels of the genotypes. The observed average heterozygosity (0.24) was less than the expected heterozygosity (0.69). Similar finding was also observed by Lu et al. (23) in the genotypes from China. This could be due to lack of hybridization as the crop is has erratic flowering, lack of seed set (51), and clonal propagation. The poor natural crossing/fertility may be a result of triploidy as well as its localization to backyard places. Chaïr et al., 2016 (52) observed over 74.2% population of the taro from India were triploid, majority of which (72%) were collected from the Meghalaya state itself.

The results of analysis of molecular variance (AMOVA) have shown the presence of 89.57% of diversity within the population which also supported our findings of less hybridization possibilities (Table 6). Further, cluster analysis based on SSR genotyping (Figure 6) has also shown wider diversity among the genotypes of aroid species. All the genotypes were grouped into 3 major groups, Cluster-I (21 genotypes) and II (12 genotypes) were comprised of popular cultivars and landraces collected from the other parts of the country, while the genotypes of Garo Hills such as *Colocasia fallax* cv. Chigi a leafy type taro, Tajiting Purple and Tagiting White (*Xanthosoma* spp.) grouped in Cluster-III. Genotype, Ganima (*Colocasia gigantia*) cultivated for the petioles and leaves was found to be most diverse form rest of the species. Moreover, *Colocasia fallax* cv. Chigi a leafy, dwarf type and shade-loving, accession found wild and also cultivated in kitchen garden for their year-round leaves and petioles was closer to local landrace Takiltom and Tama. Similarly, under principal coordinate analysis, the landraces were distributed with genotypes from different geographic origins. Similar findings were also observed in the genotypes of taro in Pacific Ocean island, Vanua Lava, Vanuatu (53). The cluster analysis, also indicated the presence of the higher genetic variation within the local genotypes of project site (Rombage, Meghalaya), which are mainly known by their local name like, Rengama and Tasakrek each with two genetically distinct variants, i.e., Rengama-1, Rengama-2 and Tasakrek-1, Tasakrek-2, respectively.

The genotypes were differentiated into 2 genetic groups with maximal ΔK at K = 2 according to the analysis of genetic structure of the 33 microsatellite markers. The 58.62% of the population showed admixture, and it was more common among the popular cultivars and landraces collected from other parts of the country (Figure 8). The least admixture was observed in other aroid species including *Xanthosoma* spp. (1 & 8) and *Colocasia esculenta* var. *aquatilis* (23), which might be attributed to poor natural crossing/fertility. Further, pairwise genetic divergence between the groups were also from low (0.03) to high (0.80) F*_ST_* value. Values between 0.00–0.05 indicate little divergence, 0.05–0.15 moderate divergence, 0.15–0.25 high divergence, and over 0.25 a very high degree of divergence (40). The maximum differentiation (Figure 9) was observed between *Colocasia fallax* cv. Chigi (leafy type) to Tharsing (*Colocasia esculenta* var. *aquatilis*.) and *Colocasia fallax cv.* Chigi (leafy type) to Ganima (*Colocasia gigantea*), and Ganima (*Colocasia gigantea*) to tannia (*Xanthosoma* spp). The high degree of genetic differentiation could be due to restricted gene flow between the populations, evolution in different ecology of the region, and somaclonal variations. Moreover, the least differentiation was observed between eddo (*Colocasia esculenta* var. *antiquorum*) and dasheen type (*Colocasia esculenta* var. *esculenta*) genotypes. The stolon farming landrace Tharsing (*Colocasia esculenta* var. *aquatilis*.) was found closer to dasheen/bunda type species (*Colocasia esculenta* var. *esculenta*) with the least differentiation, and this could be due to evolution and selection of the dasheen type genotypes form the stolon farming landraces. Mathews (54), also reported that the cultivated taro originated from the *Colocasia esculenta* var. *aquatilis* through natural differentiation and human selection in tropical areas from northeast India to Australia/New Guinea. However, comparatively least differentiation has been observed in the core set having multi-corm, and cormel genotypes of different geographical origin in China (55).

The above findings of the present investigation have shown the presence of wider genetic diversity in aroids species in the region. The population has low heterozygosity and wider genetic diversity at the molecular level. The superior genotypes can be selected based on consumer demands for the size or weight of the corms/cormels, as they are governed by additive gene action and responsive to selection. The present study has identified landraces at the project site with unique ethnobotanical uses and that have certain nutritional values, like genotypes rich in total oxalate used for cooking with only meat, high starch and low to medium oxalate for breakfast, and genotypes higher in sugar content and low in acridity for baby food need to be conserved and utilized for sustainable *Jhum*/shifting farming, enhancing nutritional security, and strengthening future crop improvement programs. Moreover, in addition to common eddo and dasheen type genotypes, more taxonomic studies on other groups of cultivated aroids, especially fused type eddo and dasheen, as well as leafy type are required. Further, this study also advocate for nutritional profiling, especially genotypes grown for the leaf and petioles purpose in the region as they also have alternative uses in feed ingredients for rearing of pigs, ducks, and poultry.

## Data availability statement

The raw data supporting the conclusions of this article will be made available by the authors, without undue reservation.

## Author contributions

VV conceptualize the study and prepared the manuscript with contributions from co-authors. VV, HT, and NS acquired the fund for the research work. VV and PC carried the field trails and data collection, biochemical, and molecular analysis. AK and NS carried the data analysis. All authors approved the final version.

## Conflict of interest

The authors declare that the research was conducted in the absence of any commercial or financial relationships that could be construed as a potential conflict of interest.

## Publisher’s note

All claims expressed in this article are solely those of the authors and do not necessarily represent those of their affiliated organizations, or those of the publisher, the editors and the reviewers. Any product that may be evaluated in this article, or claim that may be made by its manufacturer, is not guaranteed or endorsed by the publisher.

## Footnotes

^1^ http://taylor0.biology.ucla.edu/structureHarvester/
