# Supplementary material for: Assessment of ethnobotanical uses, household, and regional genetic diversity of aroid species grown in northeastern India
Source: Front Nutr. 2023 Mar 31;10:1065745. doi: 10.3389/fnut.2023.1065745 (PMC10102657; doi:10.3389/fnut.2023.1065745)
Supplement: Supplementary file 1 [file Table_1.DOCX]

| SUPPLEMENTARY TABLE 1. Performance of Aroid species for growth, yield and quality traits | | | | | | | | | | | |
| --- | --- | --- | --- | --- | --- | --- | --- | --- | --- | --- | --- |
| Genotypes | Plant  height  (cm) | Petiole  length  (cm) | No side  shoots | Average Corm weight (g) | Average Cormel Wt.(g) | Yield  (kg/plant) | Yield (t/ha) | Total Sugar (%) | Starch  (%) | Oxalic acid  (%) | Dry matter  (%) |
| ML-1 | 145.29 | 105.33 | 4.84 | 167.00 | 56.00 | 1090.59 | 22.21 | 2.48 | 20.05 | 0.16 | 23.08 |
| ML-2 | 114.90 | 87.02 | 5.67 | 178.67 | 36.61 | 1105.00 | 23.00 | 4.08 | 26.73 | 0.11 | 25.08 |
| ML-9 | 120.75 | 91.75 | 4.17 | 165.67 | 39.22 | 935.18 | 18.41 | 3.90 | 17.65 | 0.20 | 21.91 |
| Arcol -1 | 103.49 | 69.82 | 3.11 | 180.00 | 37.16 | 925.83 | 17.75 | 2.89 | 23.23 | 0.19 | 23.08 |
| Arcol-2 | 118.29 | 94.33 | 4.34 | 188.33 | 46.00 | 734.10 | 15.10 | 4.94 | 21.28 | 0.12 | 20.25 |
| Arcol-3 | 121.45 | 96.67 | 4.84 | 83.33 | 20.18 | 286.54 | 8.07 | 2.72 | 20.40 | 0.16 | 21.25 |
| Arcol-5 | 113.85 | 83.50 | 3.34 | 205.83 | 48.06 | 1153.40 | 22.70 | 2.18 | 17.55 | 0.18 | 24.08 |
| Arcol-6 | 110.35 | 82.53 | 4.09 | 418.89 | 68.14 | 1127.70 | 19.24 | 3.13 | 22.98 | 0.28 | 25.08 |
| Arcol-7 | 126.59 | 110.64 | 4.92 | 600.00 | 75.68 | 1086.67 | 18.15 | 3.61 | 21.18 | 0.18 | 18.25 |
| Muktakeshi | 101.41 | 79.30 | 5.00 | 132.50 | 33.04 | 975.00 | 22.24 | 4.55 | 17.95 | 0.13 | 25.08 |
| Meghalaya Local | 119.73 | 99.60 | 1.84 | 432.56 | 14.87 | 624.05 | 13.03 | 2.87 | 23.09 | 0.20 | 21.08 |
| Meghalaya Coll-1 | 125.14 | 105.24 | 3.70 | 358.06 | 28.58 | 959.59 | 20.44 | 2.33 | 18.16 | 0.22 | 20.58 |
| Meghalaya Coll-2 | 125.25 | 105.00 | 3.27 | 245.67 | 35.50 | 1085.60 | 22.19 | 3.23 | 16.66 | 0.19 | 18.08 |
| Kandha Local | 108.73 | 85.55 | 4.25 | 146.04 | 36.83 | 800.08 | 17.33 | 3.25 | 21.95 | 0.18 | 24.41 |
| Kandha-5 | 134.77 | 113.74 | 4.11 | 471.11 | 30.68 | 1162.69 | 20.38 | 4.53 | 32.49 | 0.21 | 19.75 |
| B.K. Coll-1 | 104.29 | 86.15 | 4.11 | 110.22 | 31.91 | 471.28 | 10.60 | 3.06 | 20.49 | 0.22 | 20.25 |
| B.K. Coll-2 | 95.47 | 78.14 | 2.78 | 173.44 | 33.66 | 812.64 | 17.49 | 3.21 | 15.41 | 0.11 | 17.41 |
| BCC-1 | 101.15 | 84.75 | 5.75 | 38.04 | 18.72 | 237.50 | 8.71 | 3.11 | 20.72 | 0.19 | 21.41 |
| BCC-11 | 126.44 | 102.78 | 3.05 | 86.33 | 23.05 | 775.53 | 17.41 | 3.65 | 15.48 | 0.24 | 18.75 |
| BCC-1A | 152.36 | 121.86 | 5.07 | 145.00 | 16.83 | 290.00 | 10.20 | 1.99 | 22.02 | 0.20 | 18.58 |
| KCA-1 | 96.95 | 79.50 | 3.34 | 283.89 | 17.41 | 658.41 | 14.57 | 2.27 | 16.12 | 0.16 | 27.08 |
| SJ-1 | 111.99 | 78.67 | 4.38 | 199.33 | 41.90 | 1234.93 | 25.19 | 2.50 | 29.06 | 0.10 | 20.41 |
| TMV-293 | 114.92 | 86.13 | 3.90 | 147.22 | 39.90 | 779.24 | 15.73 | 3.91 | 15.32 | 0.17 | 22.08 |
| C-149 | 89.35 | 67.24 | 4.22 | 148.33 | 32.78 | 696.08 | 14.57 | 4.37 | 21.76 | 0.19 | 18.91 |
| IG Coll-5 | 137.55 | 108.54 | 3.20 | 100.00 | 24.50 | 636.02 | 13.41 | 2.84 | 23.51 | 0.24 | 16.75 |
| Telia | 117.63 | 91.39 | 2.84 | 99.67 | 37.03 | 1072.61 | 22.03 | 2.79 | 21.50 | 0.20 | 18.41 |
| Panchmukhi | 167.62 | 141.19 | 1.95 | 633.33 | 20.16 | 1620.56 | 27.67 | 3.95 | 22.34 | 0.38 | 27.08 |
| Sunajuli | 108.51 | 83.72 | 3.78 | 149.44 | 38.33 | 1036.02 | 20.92 | 2.50 | 26.53 | 0.16 | 22.41 |
| Nainital | 118.99 | 96.50 | 2.89 | 165.67 | 32.75 | 897.00 | 17.99 | 4.90 | 21.97 | 0.20 | 25.08 |
| White Gouriya | 140.29 | 117.48 | 4.17 | 115.00 | 34.13 | 1329.75 | 27.53 | 2.39 | 30.32 | 0.19 | 23.91 |
| Nadia Local | 127.55 | 109.55 | 3.64 | 114.50 | 45.38 | 969.53 | 19.75 | 3.12 | 25.03 | 0.26 | 20.75 |
| Kadina Local | 127.17 | 105.86 | 4.75 | 116.67 | 59.36 | 1338.10 | 26.80 | 1.93 | 21.22 | 0.19 | 19.58 |
| Naga Local | 114.41 | 69.12 | 4.50 | 375.00 | 33.14 | 666.67 | 14.28 | 3.89 | 21.56 | 0.17 | 26.18 |
| C-3 | 124.20 | 68.92 | 3.56 | 683.33 | 59.60 | 1544.17 | 25.79 | 4.05 | 23.77 | 0.15 | 24.08 |
| Naya Bunglow | 122.54 | 71.15 | 3.00 | 238.89 | 39.26 | 720.83 | 14.71 | 3.14 | 21.04 | 0.18 | 22.98 |
| Tajiting purple | 111.89 | 77.90 | 4.00 | 511.11 | 28.20 | 1133.33 | 22.61 | 2.74 | 24.08 | 0.23 | 19.50 |
| Takilltom | 104.03 | 62.12 | 2.89 | 125.00 | 21.25 | 676.67 | 13.28 | 3.10 | 17.67 | 0.25 | 24.58 |
| Tamachok | 104.27 | 67.92 | 3.11 | 244.44 | 27.49 | 841.67 | 17.47 | 2.82 | 22.35 | 0.20 | 21.18 |
| Thranga | 109.79 | 72.15 | 3.00 | 215.56 | 35.97 | 708.33 | 14.82 | 3.60 | 21.00 | 0.24 | 22.58 |
| Tajiting white | 108.61 | 67.60 | 3.11 | 222.22 | 18.41 | 566.67 | 12.15 | 3.48 | 20.65 | 0.24 | 17.60 |
| Rengama | 206.04 | 145.08 | 2.78 | 468.33 | 48.29 | 1441.67 | 27.00 | 3.38 | 22.11 | 0.18 | 20.78 |
| Tamittim | 105.29 | 61.47 | 2.56 | 180.00 | 44.37 | 537.50 | 12.08 | 3.47 | 22.98 | 0.20 | 23.38 |
| Tamachongkham | 110.10 | 67.38 | 3.44 | 217.78 | 130.00 | 1833.33 | 31.40 | 2.78 | 19.86 | 0.82 | 21.88 |
| Thangling/ | 98.58 | 65.73 | 3.11 | 205.56 | 40.97 | 995.83 | 20.52 | 2.88 | 23.99 | 0.22 | 20.58 |
| Tamagitang/Tamangsing | 105.67 | 66.10 | 2.56 | 348.89 | 58.72 | 1258.33 | 25.69 | 2.53 | 20.96 | 0.21 | 19.20 |
| Tamakam/Tama | 97.80 | 73.87 | 2.56 | 222.22 | 71.67 | 1466.67 | 30.15 | 4.19 | 21.96 | 0.42 | 20.58 |
| Thangitang/Tharsing | 108.14 | 66.13 | 4.89 | 163.89 | 76.00 | 461.67 | 10.45 | 2.40 | 19.71 | 0.17 | 19.05 |
| Teka/Takongki | 97.98 | 70.07 | 3.00 | 138.89 | 20.55 | 376.67 | 9.01 | 4.30 | 20.55 | 0.16 | 20.58 |
| Tasakrek-1 | 107.33 | 86.67 | 3.33 | 281.67 | 37.00 | 774.33 | 16.88 | 4.17 | 25.37 | 0.12 | 23.40 |
| Ganima | 119.07 | 101.00 | 2.10 | 222.33 | 84.33 | 493.33 | 8.34 | 2.93 | 23.87 | 0.28 | 21.26 |
| Rengama-2 | 141.33 | 107.67 | 4.50 | 435.00 | 348.33 | 1400.00 | 26.39 | 3.50 | 23.17 | 0.23 | 23.03 |
| Rongrem | 129.67 | 114.00 | 4.33 | 350.00 | 270.00 | 1383.33 | 28.10 | 3.00 | 23.33 | 0.23 | 20.23 |
| Selection C-3 | 133.73 | 103.83 | 5.92 | 550.00 | 45.94 | 1044.44 | 18.02 | 3.26 | 17.20 | 0.16 | 25.25 |
| Mean | 118.65 | 89.35 | 3.73 | 249.05 | 50.83 | 928.92 | 18.68 | 3.26 | 21.65 | 0.21 | 21.66 |
| SE(m) | 2.33 | 1.52 | 0.27 | 20.15 | 2.29 | 43.96 | 0.43 | 0.09 | 0.45 | 0.04 | 0.23 |
